# Secondary stressors and their psychosocial impacts on healthcare staff: lessons from a qualitative systematic review from the COVID-19 pandemic in the UK

**DOI:** 10.1192/bjo.2025.51

**Published:** 2025-05-19

**Authors:** Evangelos Ntontis, Richard Williams, Katarzyna Luzynska, Abigail Wright, Anastasia Rousaki

**Affiliations:** School of Psychology and Counselling, The Open University, Milton Keynes, UK; Welsh Institute for Health and Social Care, University of South Wales, Pontypridd, UK; School of Psychology and Counselling, The Open University, Milton Keynes, UK; Institute for Clinical and Economic Review, Boston, MA, USA; School of Health Sciences, University of Manchester, Manchester, UK

**Keywords:** COVID-19, extreme events, stress, secondary stressors, healthcare

## Abstract

**Background:**

Extreme events (e.g. floods and disease outbreaks) can overwhelm healthcare workers (HCWs) and healthcare systems. During the COVID-19 pandemic, high levels of distress and mental ill health were reported by HCWs.

**Aims:**

To examine and synthesise research findings reported in the qualitative literature regarding the stressors, and their psychosocial impacts, faced by HCWs in the UK during the COVID-19 pandemic, and to provide lessons for future support.

**Method:**

Qualitative articles were identified in EMBASE and OVID (preregistered on PROSPERO: CRD42022304235). Studies were required to have been published between January 2021 and January 2022 and to have examined the impact of COVID-19 on UK HCWs. We included 27 articles that represented the experiences of 2640 HCWs, assessed their quality using National Institute for Health and Care Excellence criteria and integrated their findings using thematic synthesis.

**Results:**

Several secondary stressors were identified, including lack of personal protective equipment, ineffective leadership and communication, high workloads and problems stemming from uncertainty and a lack of knowledge. Stressors were related to adverse psychosocial outcomes including worry, fatigue, lack of confidence in oneself and senior managers, impacts on teamwork and feeling unappreciated or that one’s needs are not recognised.

**Conclusions:**

Our thematic synthesis moves beyond simply mapping stressors faced by HCWs by considering their antecedents, origins and psychosocial impacts. Utilising a theoretical framework that points towards systemic deficiencies, we argue that secondary stressors can be modified to remove their negative effects. Consequently, workforce planning should shift from focusing on individual change towards amending psychosocial environments in which HCWs work.

The COVID-19 pandemic, caused by the SARS-CoV-2 virus, led to large numbers of people being infected and high death rates across the globe. Healthcare workers (HCWs) were particularly affected because they were exposed to the virus through their work with infected people, in addition to their exposure as members of the public.^1^ Also, they witnessed other people being severely sick or dying and were working in extremely demanding environments. Thus, it is not surprising that multiple syntheses of evidence point to a high prevalence of reported symptoms of fatigue, distress, depression, anxiety, post-traumatic stress disorder and reduced well-being.^1–6^ Our aim in this qualitative systematic review is not to reiterate what previous researchers have shown regarding the psychosocial and mental health tolls taken by the COVID-19 pandemic on healthcare workers in the UK as in other countries; rather, we wish to move one step further and consider the antecedents, origins and psychosocial impacts of the stressors faced by healthcare staff during the early stages of the pandemic in the UK, and to reflect on lessons for future support during adverse challenges.

## Impacts of the COVID-19 pandemic on healthcare staff

Existing research has highlighted the psychosocial impacts of the COVID-19 pandemic on healthcare staff across the world. Two meta-analyses of the impact of the pandemic on HCWs showed that a large percentage reported experiencing symptoms of depression, anxiety and insomnia.^3,5^ Similar systematic meta-analytic examinations of the mental health problems experienced by HCWs showed that they reported experiencing symptoms of post-traumatic stress disorder, anxiety, depression, insomnia, burnout and distress.^2,4^ These effects can be exacerbated by particular risk factors such as being female, working on the front line, being a nurse, being younger, having less social support or experiencing more occupational stressors (e.g. significant workloads, lacking protective equipment, working in shared spaces) among others.^1,3,5,7^ These results are far from surprising. In general populations that experience extreme events, people with higher levels of exposure, women, ethnic minorities, poorer populations and people with fewer psychosocial resources, or those with previous experiences of mental health problems, are more likely to demonstrate adverse psychological outcomes.^8^

These findings show clearly that adverse psychosocial experiences are influenced by factors other than disaster exposure alone. For this reason, Norris and colleagues^8^ list secondary stressors as one of the risk factors associated with the prevalence of psychosocial problems. On the one hand, primary stressors are inherent in extreme events, including viral infections, floodwaters or fires,^9^ and are associated with direct exposure to such incidents. On the other hand, secondary stressors refer either to either people’s pre-existing life circumstances and social factors (e.g. policies, practices and social, organisational and financial arrangements) that become impactful during an extreme event, or inefficient or problematic societal and organisational responses to the extreme event.^10,11^ Examples of secondary stressors in extreme events include problems in claiming insurance payments, insufficient housing, miscommunication, poor living and working conditions that persist and people’s disconnection from healthcare and other services on which they rely.^12–15^ Secondary stressors can cause significant distress, persist for a long time and increase demands for community and institutional support.

## The workforce context in the UK

Considering that secondary stressors primarily reflect organisational deficiencies and ineffective responses to extreme events that can harm workers, we briefly describe the situation affecting the healthcare workforce in the UK and how it can promote distress. Beneficial scientific advances in healthcare have contributed to a chronic imbalance between supply and demand in a setting of severe budgetary limitations. This is, if anything, worsening.^16^ The National Health Service (NHS) workforce has faced chronic strain over many years, with workload pressures continuing to grow and the imbalances becoming progressively harder to meet, particularly in the last decade.^17^

Before the pandemic, retention, recruitment and mental health challenges were exacerbating long-term problems with working conditions.^18^ However, it is also evident that some of the causes of pressure were not only fiscal but resulted from stressors that were not adequately recognised or dealt with. In 2018, a report by the UK’s General Medical Council presented evidence regarding the sources of pressure faced by doctors, pointing towards issues related to workload, staffing, work–life balance and lack of support among others.^17^ The same report stated that the NHS was ‘at a critical juncture’ (p. 24). The following year, a report by the British Medical Association^19^ pointed to similar stressors faced by healthcare staff including poor work–life balance, understaffing, lack of time for professional development, inability to provide care perceived as adequate, problematic hierarchies and bullying, or lacking basic amenities and adequate breaks among others. The aforementioned problems persisted during the COVID-19 pandemic and, particularly during its earlier stages, intensified the impact on HCWs’ well-being, stress, fatigue and burnout. More recently, Oeppen et al have called for the NHS to do more in the future to prevent fatigue in healthcare staff, because their needs have not ended or stopped rising as the additional pressures from COVID-19 have reduced.^20^

Reflecting on lessons for the future is important, for at least three reasons. First, it was not the COVID-19 pandemic that first created unsustainable demands in the UK’s healthcare system; the NHS had been under huge strain for a long time before the pandemic emerged, and various stressors faced by HCWs were already prevalent.^21^ Second, many of the stressors experienced by healthcare staff resulted from suboptimal responses to the pandemic by governments and/or healthcare systems at the point when the pandemic emerged and during subsequent waves. Third, considering that the climate crisis is increasing the frequency and intensity of extreme events,^22,23^ as well as the recent dismantling of public healthcare services in neoliberal economies,^24,25^ healthcare systems and the staff working in them are very likely to face more demands and strain in the future. What all three points share is that the stressors described are not only tractable due to their being rooted in particular systems and practices, but that they are also amenable to change through both pre-disaster preparedness activities and adequate institutional responses when incidents occur.

## The study reported in this paper

Attempts to address HCWs’ well-being and psychosocial and mental healthcare needs^18^ take place within a context of severe and long-lasting impacts of systemic and institutional deficiencies. Thus it has become clear that what is necessary continues to be a stronger focus on how particular institutional structures exacerbate and sustain distress but also, more positively, on how social support and the physical, psychosocial and moral environments within which healthcare workers have found themselves can buffer negative experiences.^26–28^

Based on the above, our aim in this paper is to look more closely into the psychosocial impacts of particular organisational arrangements and responses in the NHS. This is crucial if HCWs are to be supported adequately in both routine care and future extreme events.

We report findings from a systematic review developed from a broader project commissioned by NHS England, which involved scoping the literature published in the UK between 2021 and early 2022 regarding the impacts of COVID-19 on healthcare workers during the earlier stages of the pandemic. The consistently high quality of the qualitative papers, and the overarching theoretical significance of their findings, led us to develop and conduct the preregistered qualitative systematic review that we present here.

## Method

### Search strategy and selection criteria

The design and reporting of our review were informed by the Preferred Reporting Items for Systematic Reviews and Meta-Analysis (PRISMA) statement (http://www.prisma-statement.org/). Our review was registered with, and met the criteria set by, PROSPERO, the international prospective register of systematic reviews (CRD42022304235; registration accepted 31 January 2022). PROSPERO accepts registrations of systematic reviews before commencement of data screening or extraction. Consequently, the databases were test searched first on 18 January 2022 and then on 29 January 2022.

Author A.W. searched OVID and EMBASE, two major scientific databases in the social, psychological and health sciences. Our search terms included variations of certain keywords, including: COVID-19; wellbeing; distress; psychological; psychosocial; mental health; staff; doctor; allied health; nurse; NHS; social care; consultant; medical staff; and United Kingdom. We included only articles published in English that referred to the UK and that were published between January 2021 and January 2022. Non-empirical reports, grey literature, blogs or opinions pieces were excluded. The keywords used as search terms in the databases were tailored appropriately for each database.

### Selection procedure, screening, data extraction and quality assessment

**Fig. 1:**
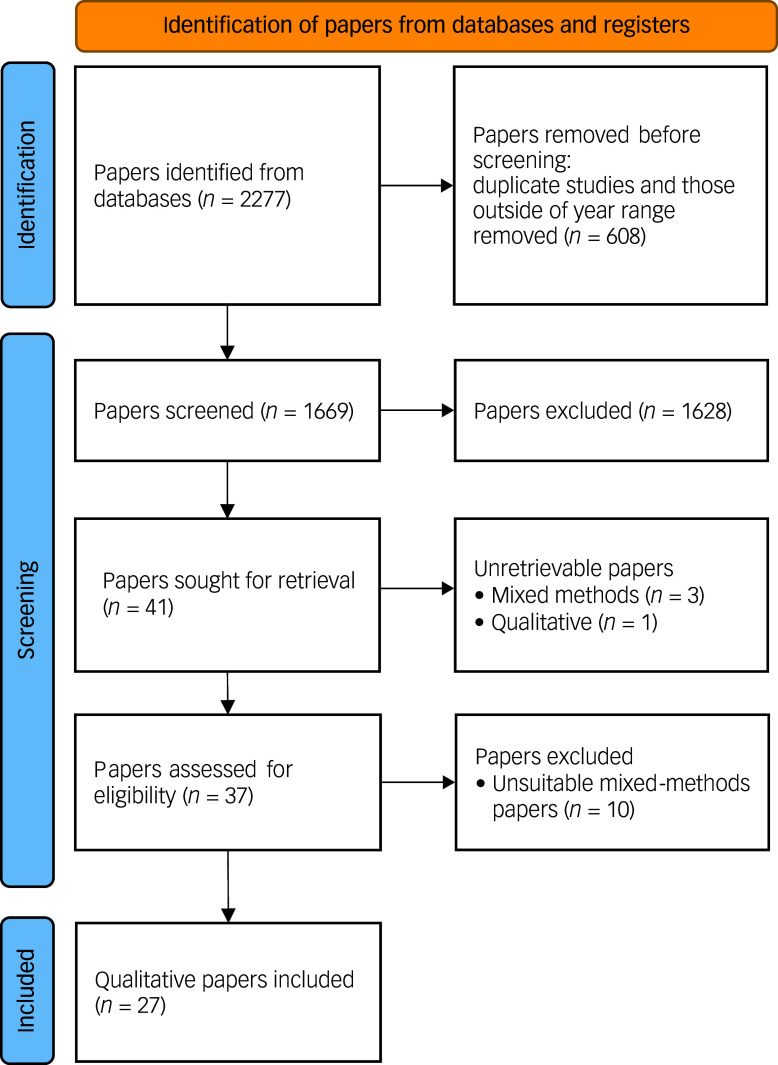
PRISMA flow diagram of search and selection process. PRISMA, Preferred Reporting Items for Systematic Reviews and Meta-Analysis.

The literature search returned 2277 studies, the titles, abstracts and other supporting information of which were entered in a spreadsheet. Of these, 437 were duplicate records and 188 studies were published outside the pre-specified year range (there was some overlap between those two categories) and, in total, we removed 608 records before the screening stage. Subsequently, two authors (K.L. and E.N.) screened the titles and abstracts of the remaining 1669 articles, excluding those not relevant to our aims (e.g. papers reporting quantitative studies). This led to the removal of 1628 papers. In cases of doubt, the procedure was for one reviewer to discuss with the other reviewer and, if disagreement occurred, a third author (R.W.) was to be consulted. The latter course was not required. The screening process started on 2 February 2022 and we extracted the data on 8 March 2022. Following the screening stage, we proceeded to data retrieval. At this stage, we sought to retrieve both qualitative and mixed-methods papers, because we found that the latter include a qualitative aspect. We sought to retrieve 41 papers but could not retrieve 4 of those; 1 was qualitative and 3 used mixed methods. Our overall search and selection process is shown in Fig. 1.

Following reinspection of the literature included in this study in mid-2024, we were able to identify one previously unretrievable qualitative study (a preprint that was eventually published around 7 months after our database search took place) and one mixed-methods study. However, we decided not to include these papers because they had not been published when our search was taking place, and therefore lay outside the terms of the protocol. Nevertheless, to mitigate against selection bias, we assessed these papers in light of the analysis reported below and did not identify any contradictory findings.

We assessed the 37 papers that we retrieved. We decided to not include 10 papers reporting mixed-methods studies because those did not report in-depth analyses of the qualitative data and did not satisfy the quality criteria reported below. Many reported the results of quantitative questionnaires, which included open-ended questions at the end and, in some cases, presented either isolated quotes or descriptive summaries of these quotes. However, they did not reach the depth required for stand-alone qualitative analyses. Nevertheless, we did examine separately the qualitative aspects of these mixed-methods papers and found that their findings were in line with the results we report in this paper.

For transparency, the ten mixed-methods papers are listed in Supplementary Materials. We have no reason to believe that not including them has altered our findings.

We used the quality appraisal checklist endorsed by the National Institute for Health and Care Excellence (NICE)^29^ to assess the quality of the 27 qualitative papers that comprised the final data-set of this systematic review. NICE has a range of criteria against which it assesses research studies, such as the appropriateness of a qualitative design, clarity of study aims and data collection processes, thorough descriptions of source population, rigour of the analysis and richness of the data presented, reliability of the analysis, appropriateness of conclusions and their grounding on the data, ethical issues, quality control and reflexivity among others. All papers were assessed by three team members (K.L., E.N. and A.R.) and were given a score based on the these criteria. There were no disagreements among the team members. Most papers were judged as being of very high quality because they fullfulled the suggested criteria. We identified some minor limitations in four papers (papers m, t, y, aa in Table 1; e.g. the discussion section in one paper did not adequately reflect the results, and some papers did not include reflexivity statements). When considering whether to disregard these papers, we took into account other strengths (e.g. well-written analyses, clear aims and sampling procedures, quotes used to illustrate their points) as well as whether the findings were in line with other studies; we thus decided to keep those in the final synthesis. A list of papers, their aims and characteristics and quality control measures is given in Table 1.

**Table 1:** Papers reporting qualitative studies that were reviewed systematically

Paper (letter as it appears in the analysis)	Author (number in reference list), year	Focus of study reported in the paper	Sample and recruitment	Period of data collection	Data collection method	Data analysis approach/methodological orientation	Theoretical perspectives guiding research questions and interpretations of findings	Reporting of quality control measures
a	Al Ghunaim et al^31^ 2021	Challenges of the pandemic to work and personal life, and personal impacts of stress	141 surgeons working in the NHS voluntarily responded to a survey available to all UK surgeons (apart from those retired)	May–July 2020	Qualitative, open-ended questions as part of a wider longitudinal survey	Thematic analysis	Not reported	Yes
b	Aughterson et al^32^ 2021	An examination of the psychosocial well-being of health and social care professionals working during the COVID-19 pandemic	25 participants from various front-line professions in health and social care. Purposive recruited through social media, personal contacts, newsletters and participants in another nationwide survey	May–September 2020	Semi-structured, one-to- one, telephone or video interviews	Reflexive thematic analysis	Berkman’s social networks framework and Antonovsky’s Sense of Coherence theory	Yes
c	Baldwin and George^33^ 2021	Deeper understanding of experiences of working during the pandemic, the reported impact of this work and the needs of staff and how they could be better supported	Purposive sampling of 19 qualified healthcare professionals (doctors, nurses, allied health)	March–May 2020	Semi-structured, one-to- one, face-to-face or telephone interviews	Thematic analysis informed by framework analysis	Not reported	Yes
d	Billings et al^34^ 2021	Exploration of experiences and needs of mental health professionals working to support front-line healthcare workers during the COVID-19 pandemic	28 mental health professionals recruited purposively through Twitter and through snowballing via mental health colleagues	June–July 2020	Remote semi-structured interviews	Reflexive thematic analysis	Not reported	Yes
e	Billings et al^35^ 2021	An examination of front-line health and social care workers’ experiences of psychosocial support during the pandemic	25 interviews with front-line workers in health and social care recruited purposively through Twitter and through snowballing via mental health colleagues	June–July 2020	Remote semi-structured interviews	Reflexive thematic analysis	Not reported	Yes
f	Blake et al^36^ 2021	An examination of views of Supported Well-being Centre visitors and operational staff towards COVID-19 workforce well-being provision	24 employees of an acute hospital trust in UK with access to supported well-being centres could express their interest to participing in the study	June–August 2020	Video or telephone semi-structured interviews	Framework analysis	Not reported	Yes
g	Daniels et al^37^ 2021	To use COVID-19 front-line doctors’ experiences and psychosocial care needs in order to develop empirically grounded recommendations and a coherent model of psychological care	Purposive sampling of 31 UK front-line doctors specialising in emergency medicine, anaesthetics or intensive care who had consented to be contacted as part of another study	Spring and winter waves, 2020	Video or telephone semi-structured interviews	Thematic analysis informed by framework analysis	Not reported	Yes
h	French et al^38^ 2022	An exploration of NHS staff experiences of burnout and betrayal-based moral injury	16 NHS staff members (nurses, doctors, occupational therapists, trainee clinical psychologists, a paramedic, an employment specialist and a member of senior management)	Not available	Video semi-structured interviews	Reflexive thematic analysis	Critical realism	No
i	Grailey et al^39^ 2021	An exploration of the presence of perceived stressors, psychological safety and teamwork in healthcare professionals	Purposive sampling of 49 participants (39 critical care staff [24 nurses, 9 doctors, 6 physiotherapists] and 10 emergency medicine professionals [2 nurses, 8 doctors])	July–December 2020	Online semi-structured interviews	Thematic analysis	Not reported	Yes
j	Harris et al^40^ 2021	To gain insights into the difficulties experienced by front-line doctors across successive COVID-19 pandemic waves	Content analysis of 1379 responses to a single open-ended question from a larger survey: ‘Please tell us what aspects of working in the pandemic you found particularly difficult?’	January–February 2021	Open-ended question included in a questionnaire	Content analysis	An interpretivist paradigm	Yes
k	Hoernke et al^41^ 2021	To explore front-line workers’ experiences with PPE during the pandemic	46 front-line healthcare workers, media reports (39 newspaper articles and 145,000 social media posts) and 25 government PPE policies	March–July 2020	Semi-structured telephone interviews, media reports and government policies	Framework method	Framework derived from anthropological perspectives on the material politics of epidemic responses	Yes
l	Jesuthasan et al^42^ 2021	To explore ethnic minority healthcare staff members’ lived experiences and impacts of COVID-19	13 healthcare workers from diverse ethnic minority backgrounds (11 clinical, 2 admin)	Not available	Two online focus groups	Template analysis	Constructivist qualitative research paradigm	Yes
m	Kanavaki et al^43^ 2021	Kidney healthcare professionals’ perspectives on impact of healthcare delivery changes on care quality and staff well-being	59 free-text responses to survey and 8 semi-structured interviews. Participants were invited to participate in a survey and consented to be approached for an interview	August 2020–January 2021	Free-text survey responses and semi-structured interviews	Thematic analysis	Interpretative epistemology	Yes
n	Kerins et al^44^ 2021	Impact of COVID-19 on workplace needs of internal medicine trainees in Scotland	12 workshops with 72 trainees, and interviews with 10 trainees	August–December 2020	Audio-recorded workshops and subsequent semi-structured interviews	Template analysis	ABC framework of doctors’ core needs	Yes
o	Kinsella et al^45^ 2022	To explore the psychological impact of working in the COVID-19 front line	38 front-line workers from UK (*n* = 17) and Republic of Ireland (*n* = 21), all employed in ‘essential’ and front-line’ roles in occupational sectors, including healthcare, social care, retail, logistics, emergency services and defence forces. Recruited through social and news media to complete a survey and consented to being approached for an interview	June–July 2020	Phone, semi-structured interviews	Thematic analysis	Phenomenological approach	Yes
p	Liberati et al^46^ 2021	An exploration of experiences of NHS mental healthcare workers during the pandemic	Purposive sampling of 35 members of staff from NHS secondary (inpatient and community) mental health services in England. These included psychiatrists (trainees and consultants), care coordinators, mental health nurses, clinical psychologists and psychotherapists)	June–August 2020	Telephone or online semi-structured interviews	Constant comparative method	Not reported	No
q	Martin and Hatzidimitriadou^47^ 2022	An exploration of community care staff members’ role transitions as a response to the pandemic	Purposive sampling of 6 community care staff	July–October 2020	Narrative correspondence inquiry	Paradigmatic mode	Critical realism	Yes
r	McGlinchey et al^48^ 2021	An exploration of healthcare professionals’ lived experiences during the COVID-19 pandemic	10 healthcare professionals (nursing, ambulance service, mental health, midwifery and social care) recruited through snowballing and social media	April 2020	Telephone semi-structured interviews	Interpretative phenomenological analysis	Phenomenological approach	Yes
s	Montgomery et al^49^ 2021	To explore staff experiences of working in critical care during the first wave of the COVID-19 pandemic	40 NHS staff working in critical care (21 nurses, 10 doctors and advanced critical care practitioners, 4 allied health professionals, 3 operating department practitioners and 2 ward clerks)	August–October 2020	Telephone semi-structured interviews	Rapid analysis methods	Sociological lens of ‘communities of fate’	Yes
t	Newman et al^50^ 2022	To explore the experiences and emotional strain of NHS front-line workers	Survey responses from 395 front-line workers recruited through their willingness to complete the survey	April–May 2020	Online survey responses	Inductive qualitative content analysis	Not reported	No
u	Olabi et al^51^ 2022	To explore NHS front-line staff experiences of an in-house psychological support service	5 staff members (4 nurses, 1 allied health) recruited through an advertisement released by the Trust	Not available	Online semi-structured interviews	Interpretative phenomenological analysis	Phenomenological approach	Yes
v	Plessas et al^52^ 2021	To explore front-line experiences and perceptions of urgent dental care centre staff	29 dentists and 9 dental nurses recruited through purposive sampling and snowballing	June–August 2020	Telephone or online semi-structured interviews	Thematic analysis	Phenomenological approach	Yes
w	Rees et al^53^ 2021	To explore paramedics’ experiences of providing care in Wales (UK)	A purposive sample of 20 paramedics recruited through a poster circulated on social media and by email	March–November 2020	Online semi-structured interviews	Grounded theory	Not reported	Yes
x	Saleem et al^54^ 2021	To explore the experiences of front-line Pakistani emigrant physicians working during the COVID-19 pandemic	10 front-line physicians of Pakistani origin involved in the direct care and management of COVID-19 patients, recruited through purposive sampling and snowballing	May 2020	Semi-structured telephone interviews	Thematic analysis	Phenomenological approach	Yes
y	Sandhu et al^55^ 2021	To explore the impact of COVID-19 on the well-being of a dental team in a secondary care urgent dental hub	14 focus groups with 40 participants (dental nurses, specialty doctors, specialty registrars, dental core trainees, one consultant) recruited voluntarily via email and written notices in public areas	July 2020	Focus groups	Thematic analysis	Not reported	No
z	Spiers et al^56^ 2021	To explore challenges faced by junior doctors working during the COVID-19 pandemic	A purposive sample of 15 junior doctors who reported being distressed	December 2020–February 2021	Telephone or online semi-structured interviews	Reflexive thematic analysis	Not reported	Yes
aa	Walker and Gerakios^57^ 2021	To explore NHS research staff’s experiences of redeployment	A purposive sample of 43 clinical research staff from an NHS Trust who were willing to participate in an audit questionnaire	Summer 2020	Online survey	Thematic analysis	Not reported	Yes

NHS, National Health Service; PPE, personal protective equipment.

The papers were read by three members of the team (E.N., K.L. and A.R.). K.L. and A.R. manually extracted information on the papers’ authors, year of publication, period of data collection, study aims, methodology and/or theoretical frameworks, sample and reporting of quality control. These data were imported into an Excel file and became the basis for our analysis. E.N. reread the papers independently and cross-checked the analyses against the data extracted by K.L. and A.R., to ensure consistency and accuracy.

The authors of the 27 papers had used a range of data collection approaches including interviews, focus groups and online surveys with open-ended questions. They also used a range of analytical approaches (e.g. thematic analysis, content analysis, interpretative phenomenological analysis [IPA]) and theoretical frameworks. The samples spanned the experiences of a range of health and social care disciplines including doctors, nurses, psychologists, dentists and occupational therapists.

### Thematic synthesis

We followed the approach of Thomas & Harden when conducting the thematic synthesis, by first coding the findings of the primary studies then creating descriptive themes and finally establishing analytical themes.^30^

The first stage involved inductively going through the data-set and labelling evidence of the themes and their summaries using core keywords that, on the one hand, encapsulated their meaning and, on the other, allowed us to find commonalities across the data-set (e.g. personal protective equipment [PPE] or leadership were codes used to label elements identified in the data and to differentiate them from one another). Subsequently, descriptive themes were created that identified commonalities across the various codes (e.g. fear of contracting COVID-19 or lack of PPE) that eventually incorporated the psychosocial impacts of stressors and led to our final analytical themes. Review of our initial analyses drew our attention to the number of secondary stressors described by participants. Consequently, the latter part of the analysis was further developed deductively using the concept of primary and secondary stressors as an organising framework for critical assessment of the antecedents and effects of the stressors identified. The adequacy of the themes we constructed was based on an interative process of continually reflecting on our interpretation of the data available to us in relation to the themes and conceptual frameworks employed, and ongoing refinement of the themes in relation to the aforementioned observations.

All authors had input into the analysis, and writing the paper was led by E.N. and R.W.

## Results

The papers that we reviewed in detail are listed in Table 1. In the text, we refer to these in parentheses, by the letter that appears in the left-hand column of the table.

Our analysis generated five themes, all reflecting processes that occurred during the earlier stages of the pandemic. The first theme appraises concerns and negative emotions and experiences stemming directly from COVID-19. The second theme describes challenges related to PPE, and the third addresses leadership and communication problems. The fourth theme reflects uncertainty and lack of knowledge and how these were outcomes of organisational features of staff members’ institutions, and the fifth focuses on workload problems and their psychosocial impacts.

### Theme 1: fear and worry about the direct impact of COVID-19

AIn the following, the papers we examined are denoted by letters of the alphabet. As expected, in many instances participants expressed their fear of contracting COVID-19 due to their being in an environment that exposed them to patients who tested positive for the virus (c,f,h,i,l,n,o,r,s,t,v,w,y,z). Grailey et al^39^ (i) illustrate this fear through an account from a senior staff nurse who stated that:

‘It was very much felt that the people who were going in could potentially be in harm’s way.’

A similar exemplary account from a trainee is provided by Kerins et al^44^ (n):

‘Worried about spreading it … or worried about catching it … or worried about spreading it to their family or bringing it into the hospital’.

The latter quote provides insights into the multiple layers of concerns that participants experienced and reported at the onset of the pandemic. For example, due to the nature of their jobs, participants were afraid not only of contracting COVID-19 themselves, but also of transmitting it to family members and friends, thus raising concerns about the safety of people other than themselves (a,b,e,f,l,s,v,w,y), some of whom could have underlying health problems (b,s,w). Al-Ghunaim et al^31^ (a) provide one such account from a surgeon:

‘Fear of bringing the virus home and infecting my family and my mother-in-law with lung cancer’.

The authors of some papers raised concerns regarding the psychosocial toll of HCWs witnessing so many patients being critically ill or dying (f,o,r,s,z) and witnessing front-line workers dying (f,s,w). Participants also reported fear of the unknown and a sense of uncertainty stemming from the pandemic during its earlier stages (b,I,n,t), and feelings such as anxiety (c,m,s,w,y), isolation (e,i,o,y) despair and grief (f,t,z). Kanavaki et al^43^ (m) depict the psychosocial toll of the pandemic through a doctor’s account that illustrates not only the psychological but also the social impacts of COVID-19 on the lives of health and social care workers:

‘There is anxiety relating to uncertainty and a demoralisation as so many planned activities are cancelled and contact with friends and family is reduced.’

The various elements summarised thus far provide a snapshot of the ways in which awareness of SARS-CoV-2 brought distress to healthcare workers during the onset of COVID-19 through a range of negative experiences, feelings and psychosocial impacts. Despite COVID-19 directly causing distress for participants, as is evident from the data and the wider literature, the effects of the virus were exacerbated by their interaction with a range of other stressors rooted in pre-pandemic systemic issues, as well as those introduced by certain ineffective responses to the pandemic. We present this matter in the themes that follow.

### Theme 2: problems related to personal protective PPE and their negative psychosocial outcomes

One central factor that was commonly reported as contributing to staff members’ experiences of distress was lack of access to personal PPE that raised difficulties in delivering care (a,c,f,i,k,r,t,v,x). As Baldwin and George^33^ (c) quote:

‘… police officers don’t go out without a stab vest; firemen don’t go out without wearing the full protective gear … why are healthcare staff any different? Why are we not provided with the appropriate [PPE]’.

During the onset of the pandemic, there was widespread dissatisfaction regarding the lack of PPE; its absence was not a problem directly attributable to the pandemic itself. There was both lack of preparedness and planning in the healthcare system in England in terms of having adequate stocks of PPE for times of emergency, as well as problematic organisational responses to the emergent extreme event. The latter included HCWs not being informed and therefore worrying about the quality and quantity of PPE, lacking clear guidelines on how to use PPE effectively or no accommodations being made to take into account difficulties and exhaustion arising from using PPE for prolonged periods and to adjust workloads accordingly.

In some cases, participants reported that PPE was available but they were worried about both its quality and quantity (j,k,r,w). These concerns were reported as distressing. One reason was cited by participants who acknowledged the limited nature of these resources and did not take breaks because, by doing so, they would have had to discard parts of their equipment, which was even more necessary early in the pandemic. Concerns about PPE led to increased fatigue. We present an extract from a longer quote as it appears in McGlinchey et al^48^ (r):

‘I basically have a drink of water and then put on the equipment again, because other people are waiting for their breaks, and go back in … Once you are in, you can’t really come out just to go to the toilet because it wastes PPE … you are starting to get dehydrated because you can’t drink …’

Participants also reported a lack of adoption of fit testing at the early stages of the pandemic (v,y), and feelings of fear that stemmed from an associated perceived lack of safety due to the absence of fit testing (c,w). According to a quote presented by Rees et al^53^ (w):

‘… really worried because I knew the masks didn’t fit me properly, so I was anxious and I felt a bit demotivated to be in work, that I didn’t want to be there because every day I was going in and it was a permanent risk really’.

Another related issue was the lack of training in how to properly use PPE, even when the latter was available, which made staff feel less confident in carrying out their duties in a manner that was safe for them and safe also for patients and colleagues (k,y). According to a quote presented by Hoernke et al^41^ (k):

‘haven’t had any training … some other nurses have been trained to use ventilators but there hasn’t been any PPE training or anything else at all’.

Overall, many staff did not feel protected against the pandemic during its early stages (a,f) and were afraid of transmitting it to their families and friends (f), revealing one of many potential connections between a stressor rooted in institutional and organisational problems (in this case the lack of PPE) and its interaction with the direct psychosocial effects of the extreme event (the fear of contracting the virus) that was often accentuated for members of staff from Black and minority ethnic backgrounds (f).

PPE was reported as causing personal discomfort, and this became a factor that affected staff–patient relationships and staff team dynamics. Lengthy working in PPE caused discomfort and fatigue (b,i,k,w,y), and changing from used to new equipment frequently took time and became a burden (a). Moreover, necessary use of PPE became a barrier to staff becoming familiar and communicating with other team members (i,k,s,v), made it harder for them to communicate with patients (b) and created dilemmas between effective risk infection and human contact (p) by limiting the following: visual and auditory cues; the ability to recognise and communicate with others; and coordinating and executing various tasks (a,i,k,s). Overall, PPE was reported as having a negative impact on teamwork (i). As Al-Ghunaim et al^31^ (a) quote:

‘PPE makes it difficult for patients to hear you and see your non-verbal response.’

PPE-related matters were the causes of wider problems in workplaces. Recurrently changing guidance, and the seemingly never-ending changes to levels of PPE required for different healthcare processes, did not inspire workers’ confidence in safety and senior managers (w,y). According to a quote from Rees et al^53^ (w):

‘you have been thrown information constantly, there’s updates after updates after updates, things are changing near enough I wouldn’t say hourly but frequently changing. Yes, you are probably being suitably informed, but it is overwhelming’.

Lack of organisational trust and leadership problems were common, and we consider these topics next.

### Theme 3: the multiple psychosocial outcomes of problems with leadership and communications

Problems in leadership and communications were two interconnected matters frequently reported by participants as causing significant distress among HCWs. For instance, many participants mentioned that they were unclear about rapidly changing guidelines, leading to confusion (c,j,k,o,t,v,w,y,aa). Plessas et al^52^ (v), for example, present a quote:

‘Nationally, I think things could have been done better, especially from the CDO’s [Chief Dental Officer’s] office; there were a lot of confusing messages coming out …’

A similar issue was staff receiving too much information from too many different sources without proper control over what was being disseminated, leading to staff members’ inability to trust neither the sources nor the information disseminated, or to conflicting information reaching the clinical services (e,s,t,w,aa).

Another common pattern in the studies was that of participants reporting that there was a lack of communication from staff in more senior jobs (c,g,j,p,r,v,aa), coupled with information often not being disseminated to teams (c,aa). Walker and Gerakios^57^ (aa) present a quote:

‘Difficult to keep up with changes if they are not communicated effectively’.

Overall, these patterns of findings point us towards the distressing experiences faced by staff due to poor communication from senior managers, particularly in the first and second waves of the pandemic, that became an obstacle to the former being able to carry out their jobs in an effective and safe manner.

Leadership problems and emergent organisational challenges were implicated in staff members’ negative psychosocial experiences, and especially if they were superimposed on other problems. For instance, participants reported receiving no support or feeling unsupported due to having no managers on the ground, with more senior people contacting them only through email (c,f,h,j,l,m,t). Baldwin and George^33^ (c) quote a staff member saying:

‘I have never seen any of the management people in the PPE to come in and to see what happens.’

Problems experienced as stemming from managers led to participants feeling unappreciated, undervalued and misunderstood (g,n,r,t). A quote from McGlinchey et al^48^ (r) illustrates this matter:

‘We work in a caring profession, but you don’t feel cared for.’

A sense of unfairness emerged from some participants because of their having to carry out significantly more tasks, and of having to cope with massive workloads while receiving the same salary (p,v). Disbanding rest areas and removing free parking and meals (all set up by some employers very early on) also led to some participants feeling undervalued (e,n,p). Many participants felt ignored or forgotten by managers (b,f,g). They experienced a sense of abandonment and that they were seen as disposable, throwaway resources and purely as numbers, despite managers being aware of the dangers faced by staff (c,h). French et al^38^ (h) illustrate this with a powerful quote from a nurse:

‘If I die, they don’t care. It doesn’t matter if [they] get like, you know, 600 nurses have died from COVID-19, and, you know, with higher exposure being linked to severity and things like that. And it just felt like [they] don’t care, they’ll just get somebody else in my shoes tomorrow.’

Staff also reported feeling underappreciated and not feeling listened to or involved in any decision-making processes, and on other occasions silenced (c,e,h,o,q,t,v), which was often the case for members of staff from Black and minority ethnic cultures (l). Taken together, these findings, coupled with a sense of poor decision-making by senior managers (j,aa), a perceived culture of blame from leaders and government (b), hierarchies perceived as worsening the working environment (f), as well as a pressure from managers to continue working on the front line (l), led to participants reporting fractured relationships with managers (g,h). A very powerful part of a longer quote from French et al^38^ (h) reads:

‘I feel extremely frustrated, I feel powerless, I do not feel listened to, I feel like I have nowhere to go with anything. That’s why I feel like I’ve reached the end of the road. I do feel like that.’

### Theme 4: uncertainty and lack of knowledge leading to distress and moral injury

Staff often reported problems related to their uncertainty and lack of knowledge in carrying out particular tasks, a phenomenon that was more intense during the first waves of the pandemic due to its novelty. These were distinct but interrelated experiences that caused distress. Staff were responsible for making challenging decisions (a,d,p,q,v,w,x) that were experienced as distressing. A staff member reported to Liberati et al^46^ (p):

‘I don’t have as much information to make decisions so I’m questioning my decision-making more thoroughly. I’m frightened of making the wrong decision when I’m deciding whether somebody gets a service or doesn’t get a service. That’s quite problematic.’

This matter is unsurprising and expected from people working in health-related services, because many decisions inherently involve assessing risks. However, there were clear systemic factors contributing to making various decisions particularly challenging. For instance, participants reported being asked to work in specialised areas about which they had limited or no training, expertise, or knowledge (a,d,f,n,p,q,t).

The lack of established programmes, protocols or, sometimes, equipment led to anxiety and uncertainty (d,n,q), and even participants with experience reported their lack of confidence (s). In general, many participants were worried about harming patients due to mistakes or incorrect decisions (d,p,q,t,v), especially when caring for people in critical conditions (f,p,s,w,x). An illustrative quote is presented by Rees et al^53^ (w):

‘I feel they were pushed out too quickly without adequate training and understanding from front line crews, and I fear this will lead to risky decisions being made that would not otherwise benefit the patients.’

Participants reported widespread concerns about the quality of care provided for patients, treating it as substandard (f,j,m,p,q,s,v,w,x). Moreover, staff often faced dilemmas between strict adherence to protocols and not violating the values involved in their jobs (j,p,q,w). Many informants reported experiencing self-doubt, lack of control, panic attacks, anxiety, guilt, distress or moral injury due to violation of their personal values and moral codes (f,i,j,l,p,q,s,t). Whereas distress refers to frequently expected negative experiences of stress during extreme events accompanied by particular emotions, thoughts and physical sensations, moral injury is associated with extreme emotional responses experienced when one’s moral code is violated and deeply held beliefs are transgressed.^58,59^ Grailey et al^39^ (i) quote a staff member saying that:

‘we did absolutely the best that we could possibly do, but it just in no way, shape or form was good enough. But we did what we could in the confines of our environment’.

### Theme 5: the psychosocial consequences of intense workloads

The last pattern that appears across the data-set in relation to stressors faced by health and social care workers is associated with workload problems. Many participants quoted in the papers available to us referred to intense and unsustainable workloads in extremely challenging and understaffed working environments (a,b,c,d,f,i,j,n,p,q,s,t,v,z,aa). Aughterson et al^32^ (b), for example, quote a participant stating that:

‘My routine was really like … wake up, eat something, go into work, which as shifts as nurses we had to stay in the hospital for 12 and a half hours … go home and eat something, drink something, go to sleep … then wake up and then go to work again … we have been extremely busy compared to the normality’.

Another example comes from Spiers et al^56^ (z), who quote a participant saying:

‘… on a Friday in the middle of the day when there was no consultant around […] I gained 14 new patients who I’d not met before […] that was a really stressful day’.

Across the papers reviewed, staff from different domains of care and from various specialties were described as saying that they were overburdened by a range of additional responsibilities. They opined that their hectic environment did not allow them sufficient time to care appropriately for critically ill people. They experienced a sense of stagnation due to overload because of very long shifts, and reported an inability to take breaks or think about how to best navigate the difficulties inherent in their jobs; and especially so because of the new situation they were facing.

Associated with workload problems was the fact that staff’s roles and responsibilities were changing, as well as the requirement to adapt to redeployment and new work structures (a,b,f,i,j,r,aa). McGlinchey et al^48^ (r) quote a staff member who, regarding her redeployment, said:

‘That was just dropped on us. There was no negotiations, there was no ‘these are your options, you might not have to go there’ … the thought of moving again to a different hospital almost an hour away is too much for me’.

Staff clearly thought that workloads, which they perceived as excessive, also had an impact on team performance. Sometimes, workloads led to divisions and tensions between colleagues and to breakdowns in teamwork (e,i,j,z). Being understaffed and under-resourced, for instance, led to a negative impact on team morale, which Harris et al^40^ (j) characterise as ‘shortage of staff; decreasing staff morale; cracks in the team’ which, according to the same paper, was the case for the healthcare system as a whole:

‘working in hospitals that run [at] near 100% capacity near 100% of the time (prior to the outbreak) and then expecting and trying to take a service that has little slack and stretching it further. It’s been relentless and exhausting, sometimes you are left feeling that, despite doing our best, we should be doing better but can’t, given the circumstances/resources.’

The overstretched system made it hard for colleagues to care for others due to the exhaustion they were already facing. A senior doctor reported in Harris et al^40^ (j) that:

‘My own biggest challenges have been the moral distress of watching colleagues struggle, and worrying about their well-being - this has been accentuated by the fact that my own world has been too busy in other related matters to be able to directly offload their workload, leading to [me] feeling inadequate for prolonged spells.’

## Discussion

Our aim for the study was to provide a systematic review of qualitative papers published between January 2021 and January 2022 related to the impact of the COVID-19 pandemic on UK HCWs’ experiences, which was characterised by uncertainty, high levels of infections and deaths across the globe and the absence of vaccines. Our goal was to move beyond summarising previous results and to focus on the psychosocial effects of stressors and their particular origins. We identify five main themes from our thematic synthesis. Each theme was, mainly, centred around one pattern of stressors and a diverse range of impacts stemming from it.

The first theme concerns the direct effects of COVID-19 on HCWs. Due to high levels of the SARS-CoV-2 virus present in their workplaces and elsewhere, participants were worried about being infected and/or transmitting the virus to family members, friends and acquaintances. Exposure to the virus was associated with a variable range of psychosocial outcomes including a sense of anxiety, fear, uncertainty, demoralisation and despair. These feelings coexisted with grief and were compounded by a deliberate reduction of social contacts in order to try to reduce contamination and associated feelings of isolation. These worries were not unfounded, because higher exposure, workplace settings such as shared spaces and being a front line worker were associated with increased prevalence of hospitalisation.^1,5^

In addition to concerns regarding the potent direct effects of the pandemic, participants also referred to other stressors rooted in problematic institutional practices and arrangements. For example, PPE-related problems were associated with increased rates of hospitalisation,^1^ which increases worrying and, over time, can prove to be a significant psychological burden for healthcare workers.^4,5^ Our systematic review shows that a lack of protective equipment interacted with the impact of the pandemic to intensify worry. Inability to take sufficient breaks as a result of limited access to PPE led to fatigue, dehydration, urinary infections and other health risks. Participants were worried about the quality of the PPE, and the variable conduct of fit testing led to anxiety and demotivation. Many participants reported not having training and confidence in using PPE appropriately, which increased fears of exposure to the virus, and the guidance about it changed rapidly. Using PPE also caused problems in communicating with both patients and colleagues in clinical teams.

Leadership and communication were frequently reported as problematic. Rapidly changing or excessive information, which was often not properly checked and was coming from too many sources, led to confusion and lack of trust in both messages and sources. Senior managers were reported as largely absent from the shopfloor. Hierarchies, concerns about a perceived culture of blame and lack of facilities led participants to feel unappreciated, undervalued, misrecognised and misunderstood, ignored, abandoned and seen as merely numbers. These experiences are stressors that were present from before the pandemic^19,58^ and clearly persisted, but were exacerbated and exerted their negative impacts during the pandemic. Moreover, the uncertainty, which can be inherent in relatively novel events of the magnitude of the COVID-19 pandemic, was compounded by uncertainty stemming from challenging decisions and dilemmas in the absence of appropriate training, which could lead to moral distress.^2,59^ Finally, excessive workloads due to chronic understaffing and staff sickness absences that predated the pandemic became unsustainable during the pandemic, and staff had to deal with continually changing roles and structures and stagnation and, as a result, low morale, demotivation and an impact on relationships among team members. Intense workloads and feelings of professional stagnation have also been commonly observed across large meta-analyses and have been shown to lead to negative psychosocial outcomes.^2,4,6^ It appears to us that many of the stressors represented in the themes we report were interactive and mutually reinforcing.

Apart from fear of exposure to the virus, which we regard as a primary stressor due to its being inherent in the pandemic and posing an existential threat for staff and their close others, all other problems reported in our analysis either existed before the pandemic (e.g. unsustainable workloads, understaffed services, insufficient stockpiles of PPE) or were indicators and outcomes of ineffective responses to the pandemic itself (e.g. lack of training or fit testing for PPE, invisible leaders). Thus, they can be defined as secondary stressors.^10,11^ We emphasise two points here. First, the effects of secondary stressors can be compounded because they can coexist and operate in clusters and, thereby, increase their impacts on the people affected. Second, secondary stressors can exacerbate the negative impacts of primary stressors or the pathways through which they become potent. For example, concerns regarding exposure to the virus (i.e. the primary stressor) can worsen if staff have insufficient PPE, are not trained to use it effectively and there is uncertainty regarding its appropriate use. Uncertainty and lack of knowledge can lead to decisions that might contribute to negative outcomes for patients, increasing the sense of staff feeling personally responsible and thereby exacerbating worry. Moreover, secondary stressors can amplify the effects of other secondary stressors. Being overworked can potentially lead to negative outcomes, especially in the absence of established protocols due to rapidly changing guidelines. Lack of PPE erodes trust in leaders.

Overall, the notion of secondary stressors is theoretically and practically insightful because it helps us to emphasise the systemic nature of the issues raised by participants and, thus, in regard to our ability to track these stressors and change them. In this study, secondary stressors point to occupational problems and organisational features that are problematic, with occupational stressors being particularly prevalent in our data-set. In other instances (e.g. the general public), secondary stressors reflect issues related to gender and income inequality.^62^ However, across all these cases, most of the negative experiences we report were not inevitable but rather point to problems in cultures and environments in which healthcare staff work, as well as to problems arising from the wider political decisions.^10,11^

The theoretical lens of secondary stressors is also practical because it allows us to take an in-depth look into the nature of stressors and their psychosocial impacts. This framework is useful for a number of reasons. First, it helps to establish typologies of stressors as they are identified in the existing literature and whose impacts can be prevented through timely identification and removal of each stressor. Second, this lens offers us transferability of insights. Although the systematic review we report in this paper records some of the lessons learned about meeting the needs of staff during the pandemic and other serious emergencies, it is clear that those lessons are also highly relevant in more ordinary times.

Thus, the usefulness of the notion of secondary stressors extends beyond the field of extreme events and into more ordinary work and workplace settings. Similar findings have come from within healthcare settings, with ambulance staff, for example, not only reporting distressing features of their work such as a lack of downtime, a target culture and their managers and support services not being very supportive, but also identifying gaps in their training and knowledge that would improve their working conditions and professional conditions and relationships.^63^

In our experience, secondary stressors are prone to occur throughout healthcare and most other systems, and our synthesis of the experiences of HCWs during the first and second years of the COVID-19 pandemic serves as an example through which to highlight chronic problems in the NHS that promote damaging outcomes (e.g, low staff retention, problems in recruitment).

During the decade 2010–2020, the NHS struggled with staff recruitment and retention due to chronic extreme pressure caused by inequity, inequality, funding cuts and high, persisting levels of distress and fatigue. In 2023, the General Medical Council (GMC) published research that explores the reasons why doctors have left, or may be considering leaving, the NHS to practise abroad.^64^ The report states that ‘general improvements to workplace […] could address the main reasons why doctors say they are unhappy in UK practise, and have a positive impact upon retention’. Thus, our opinion is that many of the problems with current NHS staff recruitment and retention are likely to reflect the secondary stressors faced by staff. In parallel, Maben et al review the evidence for three workplace conditions that are relevant for improving quality and safety in healthcare. They regard the key matters as: staffing; psychological safety, teamwork and speaking up; and staff health and well-being at work.^26,65^ We think that these topics have much in common with the experiences we report here regarding working conditions in the NHS during the COVID-19 pandemic. Maben et al offer helpful approaches to remedying some of the secondary stressors in healthcare services. Recent research points to the importance of social support, identity leadership and collective resilience in assisting front-line workers.^66,67^ Thus, we think that what we have learned about stressors experienced by healthcare staff during the pandemic has much to teach us about improving staff retention in the NHS. Considering the dynamics and impacts of secondary stressors can help us to think more strategically about how best to support staff and care for them, as well as better prepare for future extreme events.

Furthermore, the concept of secondary stressors could also help us to think about limitations in other systems and institutions (e.g. schools, social care and other workplaces), including how their members cope with extreme events in the face of wider ongoing social issues (e.g. poverty, discrimination).

### Lessons for future support

Thus, we argue that an exclusive emphasis on improving healthcare workers’ mental health through the development of personal resilience is likely to be inadequate unless it takes into account how organisational features and responses to extreme (and more ordinary) events affect healthcare workers. Our analysis of HCWs’ experiences and the stressors they faced during the early onset of the pandemic attests to that position and echoes findings from past serious infectious disease outbreaks.^68^ We present a list of recommendations based on our findings that can support healthcare staff facing adverse conditions:

- Adequate personal protective equipment (PPE) of high quality should be readily available.
- Staff should be trained on how to use the equipment properly, to minimise actual and perceived risks of infection and transmission.
- Managers should plan for adequate breaks, especially for staff using PPE for prolonged hours, to enable rest and reduce their fatigue.
- Managers should streamline communications and release them from specific channels to avoid conflicting and contradictory messages. These should be communicated clearly from early on, and especially so if uncertainty is expected and rapid changes occur.
- The presence of leaders on the ground is essential in order to create a sense of team cohesion around a common purpose, and so that staff feel supported and cared for. The absence of leaders can create divisions within organisations and a lack of HCWs’ sense of belonging to their institutions.
- Opinions and concerns of staff should be heard and considered by managers. This is one way to increase a sense of organisational belonging and productive vertical relations.
- Staff should be able to feel secure and supported by peers, leaders and managers when facing dilemmas and hard decisions.
- Staff should be able to feel that they are delivering appropriate levels of care in line with the moral foundations of their profession. Otherwise, they run the risk of experiencing moral distress.
- Staff should be provided with tangible support (e.g. free parking spaces, resting facilities), which is not only likely to reduce fatigue but will also increase team cohesion, trust in senior managers and cultivate feelings of being cared for and supported by their institution.
- Healthcare institutions need appropriate levels of staffing to ensure adequate rest for staff and appropriate levels of care for patients.
- Adequate state funding, infrastructure and investment in health in general are required to ensure the safety of both patients and healthcare workers, as well as adequate equipment and other resources to cope with crises.
- The recommendations we make here combine to emphasise the importance and effective planning and preparation to meet the impacts of all risks, including those of extreme events.

### Limitations

First, we oconsidered only papers published between 2021 and early 2022. Unavoidably, this means that significant papers of high quality and that might have provided novel findings that further support or contradict the findings of this review could be missing due to their having being published outside of the search periods. However, even if this is the case, we consider the risk to be minimal. Findings from research published after our search took place, and particularly from longitudinal studies conducted in the long-term aftermath of the pandemic, are in line with the findings reported here. For example, Chen and colleagues^69^ sampled healthcare workers between 2021 and 2023 and identified elevated self-reported symptoms of depression, anxiety and reduced sleep quality that were associated with a range of systemic and organisational issues, such as excessive workloads, working in the front lines or gender inequality among others. Maunder and colleagues^70^ sampled HCWs until 2023, finding no significant improvement in distress, emotional exhaustion, depersonalisation or a sense of personal accomplishment despite rates of hospitalisation decreasing. The authors point towards individual, organisational and occupational stressors that accumulated over time and depleted the workforce, suggesting the need for changes in organistional systems and cultures. Gutmanis and colleagues^71^ similarly sampled HCWs between 2021 and 2023, showing that, despite a slight reduction in emotional distress levels, more than half of HCWs were still reporting moderate to high levels of distress, indicating long-lasting negative effects. These effects were related to demographic factors (e.g. age, gender, occupational role), not being able to meet childcare needs or the operation of broader pandemic mitigation measures, with the authors pointing towards the need for organisational support. In 2024, the UK experienced a ‘quademic’ consisting of COVID-19, influenza, respiratory syncitial virus and norovirus^72^ that could have had a negative effect on the capacity of the healthcare system to act. At the same time, the longitudinal findings reported above suggest that secondary stressors remained relevant following the immediate impact of the COVID-19 pandemic. Thus we think that, despite being a limitation, our 1-year search period does not pose a significant risk for the accuracy of our results. This becomes more likely if we consider that the results reported in our sample of papers were all in the same direction, which further increases our confidence in our results. Future research should expand this line of work by exploring HCWs’ experiences and stress processes in the longer-term aftermath of extreme events, and by examining the long-term relevance of the stressors that we identified.

Second, and related to the aforementioned limitation of the search period that our review focuses on, is the issue of publication bias; however we consider this threat to our findings to be minimal. This is because, despite our sample being relatively small and focusing only on studies published in the UK and in English over a period of 1 year, our findings are in line with other qualitative and quantitative systematic reviews and meta-analyses that took place across different countries, contexts and time periods. Thus, we have no reasonable grounds to doubt the validity of our findings and their generalisability. We think that coming across contradictory findings would be relatively unexpected, considering the nature of systemic deficiencies inherent in many healthcare systems across the globe.

Third, the quality of each systematic review depends on the quality of the papers included in it. As expected, all the papers we reviewed have limitations. Nevertheless, we have tried to minimise the impact of these matters on our review. We pre-registered the review with PROSPERO and followed PRISMA and NICE guidelines on assessing the quality of the papers, the vast majority of which we judged to be of high quality. Those that did not meet this standard showed minor limitations that did not pose a risk in terms of negatively affecting our findings.

Our paper reports a qualitative systematic review of papers published in the UK regarding the effects of COVID-19 on healthcare staff. The novelty of our work lies in the use of a theoretical framework to examine the antecedents and psychosocial impacts of primary and secondary stressors, and ways to mitigate them. In the course of our analysis, it became apparent that secondary stressors were very influential in proliferating distress. Considering that recruitment and retention are continuing central concerns for the NHS, mitigation strategies should not focus so much on building individual or personal resilience in staff but rather on trying to improve workplace conditions by tracking and tackling secondary stressors so that their effects are reduced. The fact that our findings also concern problems that predated the pandemic highlights the importance of addressing occupational, organisational and other systemic deficiencies that are outside the context of extreme events. It also emphasises the importance of taking a rigorous approach to planning to meet risks of events to the staff of the NHS at national, regional and local levels.

## Supporting information

Ntontis et al. supplementary material Ntontis et al. supplementary material

## Supplementary material

The supplementary material can be found online at https://doi.org/10.1192/bjo.2025.51
